# Identification of transcriptomic signatures associated with an ac4C related gene set and candidate expression based clusters in osteoarthritis through integrative bioinformatics

**DOI:** 10.1371/journal.pone.0359336

**Published:** 2026-09-24

**Authors:** Tianyang Li, Jinpeng Wei, Hua Wu, Ming Zhang

**Affiliations:** Department of Orthopedics, Third Hospital of Shanxi Medical University, Shanxi Bethune Hospital, Shanxi Academy of Medical Sciences, Tongji Shanxi Hospital, Taiyuan, China; Public Library of Science, UNITED KINGDOM OF GREAT BRITAIN AND NORTHERN IRELAND

## Abstract

**Objective:**

To identify osteoarthritis (OA) associated transcripts overlapping a predefined N4-acetylcytidine (ac4C) related gene set and evaluate their potential as an exploratory classification signature and basis for expression based clustering.

**Methods:**

Five GEO datasets were analyzed using differential expression, functional enrichment, weighted gene co-expression network analysis, immune-signature scoring, machine learning, SHAP interpretation, consensus clustering, and gene set variation analysis. The predefined 2,135-gene set was derived from a published ac4C-RIP-seq comparison between wild-type and NAT10-deficient HeLa cells and was used only for candidate filtering. Twelve algorithms were combined into 113 two-stage feature-selection/classification pipelines, which were ranked by the mean area under the receiver operating characteristic curve (AUC) across the development cohort and two external evaluation cohorts. Five retained genes were assessed by qRT-PCR in IL-1β-treated primary mouse chondrocytes with three biological replicates per group.

**Results:**

Among 441 differentially expressed genes, eight overlapped the ac4C related set. Three pipelines shared the highest mean AUC of 0.910. The representative glmBoost–Naive Bayes pipeline achieved AUCs of 0.883 (95% CI, 0.783–0.959), 0.980 (95% CI, 0.880–1.000), and 0.867 (95% CI, 0.600–1.000) in the development cohort, GSE114007, and GSE169077, respectively. Because the two secondary cohorts contributed to pipeline ranking, these estimates represent exploratory evaluation rather than independent validation. Ultimately, five genes were retained, including *PCOLCE*, *KAZALD1*, *PDE3A*, *CRIP1*, and *ID1*. *Kazald1*, *Pde3a*, *Crip1*, and *Id1* showed nominally significant increases after interleukin-1βtreatment, whereas *Pcolce* did not. Immune signature differences and the two cluster solution were exploratory.

**Conclusions:**

A five gene OA associated transcriptomic signature linked to a predefined ac4C related gene set was identified. These findings are hypothesis generating and do not establish direct ac4C modification, independent clinical validity, or reproducible molecular subtypes.

## Introduction

Osteoarthritis (OA) is the most prevalent chronic degenerative joint disease and a leading cause of pain, disability, and reduced quality of life among the aging population worldwide [1]. Characterized by progressive cartilage degeneration, subchondral bone remodeling, synovial inflammation, and extracellular matrix (ECM) disruption, OA has traditionally been regarded as a mechanically driven disorder [2]. However, accumulating evidence indicates that OA is a complex multifactorial disease involving genetic susceptibility, aberrant inflammatory responses, metabolic alterations, and dysregulated tissue repair processes [3]. Despite substantial advances in understanding OA pathophysiology, the molecular mechanisms underlying disease initiation and progression remain incompletely elucidated, and reliable biomarkers for early diagnosis and subtype stratification are still lacking.

Recent developments in high-throughput sequencing and bioinformatics have greatly facilitated the systematic exploration of disease-associated molecular signatures. In OA research, transcriptome profiling has been widely used to identify differentially expressed genes (DEGs), altered pathways, and co-expression networks associated with cartilage degeneration and inflammatory microenvironment remodeling [4]. Nevertheless, the heterogeneity of OA and the complexity of gene regulatory mechanisms make it difficult to identify robust diagnostic markers solely through conventional differential expression analysis. Therefore, integrating multi-dimensional bioinformatics strategies, such as weighted gene co-expression network analysis (WGCNA), immune infiltration assessment, and machine learning-based feature selection, may provide a more comprehensive framework for uncovering key regulators and clinically relevant biomarkers in OA [5].

Among the various layers of epigenetic and epitranscriptomic regulation, RNA chemical modifications have emerged as important modulators of gene expression [6]. N4-acetylcytidine (ac4C) is a conserved RNA modification found in tRNA, rRNA, and mRNA, and is known to enhance mRNA stability, translation efficiency, and decoding fidelity [7]. Increasing studies have revealed that ac4C modification participates in diverse biological processes, including cell proliferation, stress response, immune regulation, and tumor progression. Dysregulation of ac4C-related regulatory networks has been implicated in several human diseases, highlighting its potential role as a novel mechanism of pathogenic gene regulation [8]. The ac4C related gene set used in this study was derived from a previously reported ac4C-RIP-seq comparison between wild type and NAT10-deficient HeLa cells [9]. Therefore, its overlap with OA associated transcripts indicates a potential association with ac4C related regulation but does not demonstrate direct ac4C modification in OA tissues. Similarly, immune signature scores inferred from bulk transcriptomic data reflect enrichment of predefined transcriptional programs rather than direct immune cell abundance. In addition to intrinsic transcriptomic dysregulation, immune microenvironment alterations are increasingly recognized as crucial contributors to OA progression. Although OA has long been considered a non-immune disease, growing evidence supports the involvement of both innate and adaptive immune cells in mediating synovial inflammation, cartilage catabolism, and tissue remodeling [10,11]. Characterizing the relationship between disease-associated genes and immune cell infiltration may therefore provide additional insight into OA pathogenesis and help identify molecular targets with immunoregulatory relevance.

We hypothesized that OA associated DEGs overlapping a predefined ac4C related transcript set could identify candidate expression signatures and exploratory OA sample groupings. We integrated five GEO datasets, performed differential expression, enrichment, WGCNA, and immune associated transcriptomic signature analyses, compared 113 machine learning pipelines, and used SHAP to interpret the retained model. The expression patterns of the five retained genes were then assessed by qRT-PCR in an IL-1β treated primary mouse chondrocyte model with three independent biological replicates. Consensus clustering and GSVA were used to explore expression based groups and pathway score differences.

The principal contribution is a transparent, hypothesis generating integration of the ac4C related transcript set with multi dataset OA transcriptomics, broad within task machine learning comparison, model attribution, immune-signature scoring, limited qRT-PCR assessment, and exploratory clustering. The study does not establish an ac4C driven OA mechanism or provide a clinically ready diagnostic test; the qRT-PCR experiment provides partial mRNA level support rather than mechanistic validation.

## Methods

### Data preprocessing

Five GEO series were analyzed. The development cohort combined GSE51588, GSE113825, and GSE117999. GSE51588 contains human knee subchondral bone samples, whereas GSE113825 and GSE117999 contain cartilage samples; the integrated matrix therefore represents mixed joint-tissue sources. GSE114007 and GSE169077 were external evaluation cohorts, and both contributed to model ranking. Platform-specific processed expression matrices were obtained from GEO, and no uniform raw-data renormalization was applied across platforms. Probe identifiers were mapped to current human gene symbols, duplicate gene entries were averaged using limma::avereps, and genes shared across the development datasets were retained. Cross-dataset batch effects were adjusted using sva::ComBat, with dataset source specified as the batch factor, a parametric empirical Bayes prior (par.prior = TRUE), and no additional biological covariate matrix. Principal component analysis was used only as a qualitative diagnostic of the adjusted matrix [12]. All analyses were performed in R version 4.3.3. Dataset composition and analytical roles are reported in S1 Table.

### Definition of the ac4C-related gene set

The predefined 2,135 gene set was derived from a previously published ac4C-RIP-seq analysis comparing wild type and NAT10-deficient HeLa cells [9]. Accordingly, this gene set represents previously reported ac4C associated transcripts rather than ac4C regulatory enzymes or OA specific ac4C modification targets. These transcripts were designated as ac4C related genes in the source study. Gene identifiers were converted to current HGNC approved symbols, and duplicate entries were removed before the overlap analysis.

### Identification of differentially expressed genes

Differentially expressed genes (DEGs) associated with OA were identified using the limma R package. Benjamini–Hochberg adjustment was used to control the false-discovery rate. Genes with |log2 fold change| > 0.585 and adjusted P < 0.05 were designated as DEGs. The cutoff of 0.585 corresponds to a 1.5-fold expression difference and was selected as a pragmatic threshold to retain moderate expression changes expected in a chronic and heterogeneous disease while maintaining false-discovery-rate significance [13].

### Functional enrichment analysis

Gene Ontology (GO) functional annotation and Kyoto Encyclopedia of Genes and Genomes (KEGG) pathway enrichment analysis were both conducted using the “clusterProfiler” R package. GO analysis provides a hierarchical classification of gene functions across three distinct categories: biological process (BP), cellular component (CC), and molecular function (MF) [14], whereas KEGG analysis primarily characterizes the coordinated regulatory networks among multiple genes involved in signal transduction and metabolic pathways [15]. By integrating these two complementary approaches, fine-grained molecular functions can be effectively linked to broader pathway-level mechanisms, thereby enabling a comprehensive functional interpretation spanning from the molecular to the systems level.

### Weighted gene co-expression network analysis

Weighted gene co-expression network analysis (WGCNA) was conducted using the WGCNA R package to identify co-expressed gene modules and examine their associations with disease status. Before network construction, genes with an across-sample standard deviation greater than 0.5 were retained as a pragmatic low-variability filter, yielding 6,061 genes. This fixed threshold was used to reduce the contribution of relatively uninformative genes; alternative variability thresholds were not systematically evaluated. A pairwise Pearson correlation matrix was constructed, and the soft-thresholding power was selected using pickSoftThreshold to approximate scale-free topology. The adjacency matrix was transformed into a topological overlap matrix (TOM) [16]. Genes were grouped by average-linkage hierarchical clustering and dynamic tree cutting, with a minimum module size of 50 genes. Similar modules were merged, module eigengenes were calculated, and Pearson correlations between module eigengenes and disease status were assessed. The module with the largest absolute correlation and lowest P-value was retained for subsequent analysis.

### Identification of ac4C-related differentially expressed genes in OA

To identify the overlapping parts of OA related transcripts with a predefined set of ac4C related transcripts, the R language package “ggvenn” was used to perform intersection analysis between DEGs and 2135 candidate gene sets, resulting in a set of ac4C related differentially expressed genes (acRDEGs). This overlapping result is only used as a candidate gene screening step and does not infer the ac4C dependent regulatory mechanism. Subsequently, the expression profiles of acRDEGs in the training cohort were visualized using the “ggpubr” R package, while their chromosomal distribution was mapped using the “CMplot” R package.

### Immune associated transcriptomic signature analysis

Single sample GSEA implemented through the GSVA package was used to calculate enrichment scores for 28 immune cell related metagene signatures originally developed in a pan-cancer context [17]. These values are termed immune cell associated transcriptomic signature scores, not cell counts or direct abundance estimates. A correlation heatmap between differentially expressed genes and immune cell populations was generated using the “reshape2” and “tidyverse” R packages. OA and control scores were compared using Wilcoxon rank-sum tests, and gene signature associations were summarized by Spearman correlation. Multiplicity adjusted immune analysis tables were not available, so the findings are treated as exploratory.

### Machine learning-based identification of hub genes

The framework included Lasso, Ridge, Stepglm, XGBoost, random forest, Elastic Net, plsRglm, GBM, Naive Bayes, LDA, glmBoost, and SVM, arranged into 113 two-stage feature-selection/classification configurations. The development matrix was centered and scaled as a whole, whereas each external cohort was centered and scaled separately. Algorithm-specific tuning or predefined parameter settings were used. Penalized regression models used 10-fold cross-validation to select lambda.min; glmBoost selected the stopping iteration using cross-validated risk, with a minimum mstop of 40; GBM used 10-fold cross-validation to select the final number of trees; and XGBoost used five-fold cross-validation to select the number of boosting rounds. Random forest was fitted with 1,000 trees and a node size of 5, whereas SVM and Naive Bayes used package-default settings without an explicit tuning grid. The global random seed was set to 123 before feature selection and model fitting. Candidate feature selection was performed on the full scaled development matrix rather than repeated within each validation fold; therefore, a fully nested, fold-contained validation procedure was not implemented. Each configuration was evaluated in the development cohort, GSE114007, and GSE169077, and the arithmetic mean of the three AUC values was used for ranking [18]. Because both external cohorts contributed to model selection, they were treated as exploratory external evaluation cohorts rather than independent holdout tests. Detailed implementation records are provided in S2 Table, and the analysis scripts are publicly available through Zenodo.

### Mouse chondrocyte culture and processing

Primary chondrocytes were isolated from the knee joint cartilage of 5-day-old male C57BL/6 mice provided by Hubei BIONT Biological Technology Co., Ltd. Neonatal mice were placed on a layer of gauze positioned over crushed ice to prevent direct contact with the ice and were anesthetized by hypothermia until the absence of the toe-pinch reflex was confirmed. Decapitation was then immediately performed using sharp surgical scissors as a secondary physical method to ensure death. All procedures were carried out by trained personnel in accordance with an approved institutional animal protocol. Following aseptic dissection, sequential digestion was performed: first with 0.25% trypsin (Boster, China) at 37°C for 30 minutes, then with 0.2% collagenase II (BioFroxx, Germany) at 37°C for 4–6 hours. Cells were then suspended in DMEM/F12 medium (HyClone, USA) supplemented with 10% fetal bovine serum (Newzerum, New Zealand) and cultured at 37°C under 5% CO_2_. Cells were passaged when they reached 80–90% confluence. For osteoarthritis induction, first- or second-generation chondrocytes were seeded into 6-well plates. Cells in the OA group were treated with 10 ng/mL IL-1β (R&D Systems, USA) for 24 hours, while the control group remained in fresh medium.

### Quantitative reverse transcription-PCR (qRT-PCR)

Total RNA was extracted using the EZNA Total RNA kit (Omega Bio-Tek, USA). First-strand cDNA was synthesized from 2 μg RNA with a Reverse Transcription kit (Toyobo Life Science, Japan). Real-time quantitative PCR was performed using SYBR Green Real-Time PCR Master Mix (Toyobo Life Science, Japan) on a CFX96 system (Bio-Rad Laboratories, USA). Relative gene expression was determined by the 2^-ΔΔCt^ method with *Actb* as the internal reference. The experiment comprised three independent biological replicates per group (n = 3). Each biological sample was measured in three technical wells, and the three Ct measurements were averaged before ΔCt and statistical analysis; technical wells were not treated as independent observations. Data are presented as mean ± SD. Control and IL-1β-treated groups were compared using an unpaired two-sided Student’s t-test, with P < 0.05 considered statistically significant. Primer sequences for Hub OA-acRDEGs are provided in S3 Table.

### Shapley Additive exPlanations analysis

Shapley Additive exPlanations (SHAP) analysis was applied to the retained fitted five-gene classification model to characterize the contribution of each gene to the model output. Grounded in cooperative game theory, SHAP quantifies the marginal contribution of each feature to individual predictions by evaluating all possible feature coalitions [19]. For each sample in the optimal model, SHAP values were computed to reflect both the magnitude and direction of each feature’s impact on the prediction output. Global feature importance was determined by ranking features according to their mean absolute SHAP values across all samples. SHAP summary plots and force plots were generated to visualize feature contributions, reveal nonlinear relationships between features and outcomes, and illustrate individual prediction explanations, respectively.

### Exploratory visualization of the five-gene classification signature

A nomogram was constructed based on the hub genes using the “rms” package in R. In this model, the “Points” axis represents the score assigned to each candidate gene, and the “Total Points” axis reflects the cumulative score of all genes. Furthermore, calibration curves and decision curve analysis (DCA) were applied to evaluate the predictive performance of the nomogram [20]. Concurrently, the “pROC” package was used to generate ROC curves for model accuracy validation—an AUC value exceeding 0.65 indicates satisfactory model performance [21]. Because these analyses were performed within the same analyzed cohort, they were considered exploratory assessments of apparent performance rather than independent validation of calibration, clinical utility, or diagnostic applicability.

### Consensus clustering of hub genes

Consensus clustering was performed using the ConsensusClusterPlus package based on the expression profiles of the five retained genes in OA samples. Candidate cluster numbers ranging from k = 2 to k = 9 were evaluated. The analysis used 50 resampling iterations, with 80% of samples and 100% of features included in each iteration. K-means clustering with Euclidean distance was applied, and the random seed was set to 123456. Consensus cumulative distribution function curves and consensus matrices were examined to determine the exploratory cluster solution [22].

### Gene set variation analysis

Gene Set variation Analysis (GSVA) is a nonparametric technique that transforms gene expression data into pathway-based enrichment scores, enabling the detection of predefined functional gene sets across samples without requiring pre-assigned classification labels. This method is widely applied in tumor heterogeneity studies, molecular classification, and biomarker identification [23]. In this study, we employed the gene sets “c2.cp.kegg.Hs.symbols” and “c5.go.Hs.symbols” from the Molecular Signature Database (MSigDB) as references. The GSVA package in R was used to evaluate pathway enrichment scores between C1 and C2 OA expression clusters, with pathways deemed significantly enriched when FDR < 0.05.

### Ethics approval and compliance

All summary statistical data used for screening Hub genes were generated from previous studies, and all original studies obtained ethical approval and personal consent. All animal procedures adhered to the ARRIVE 2.0 guidelines and were approved by the Shanxi Medical University Animal Ethics Committee (SYDL2026106), complying with the National Institutes of Health (NIH) standards for laboratory animal care.

## Results

### Identification of DEGs in osteoarthritis

**Fig 1:**
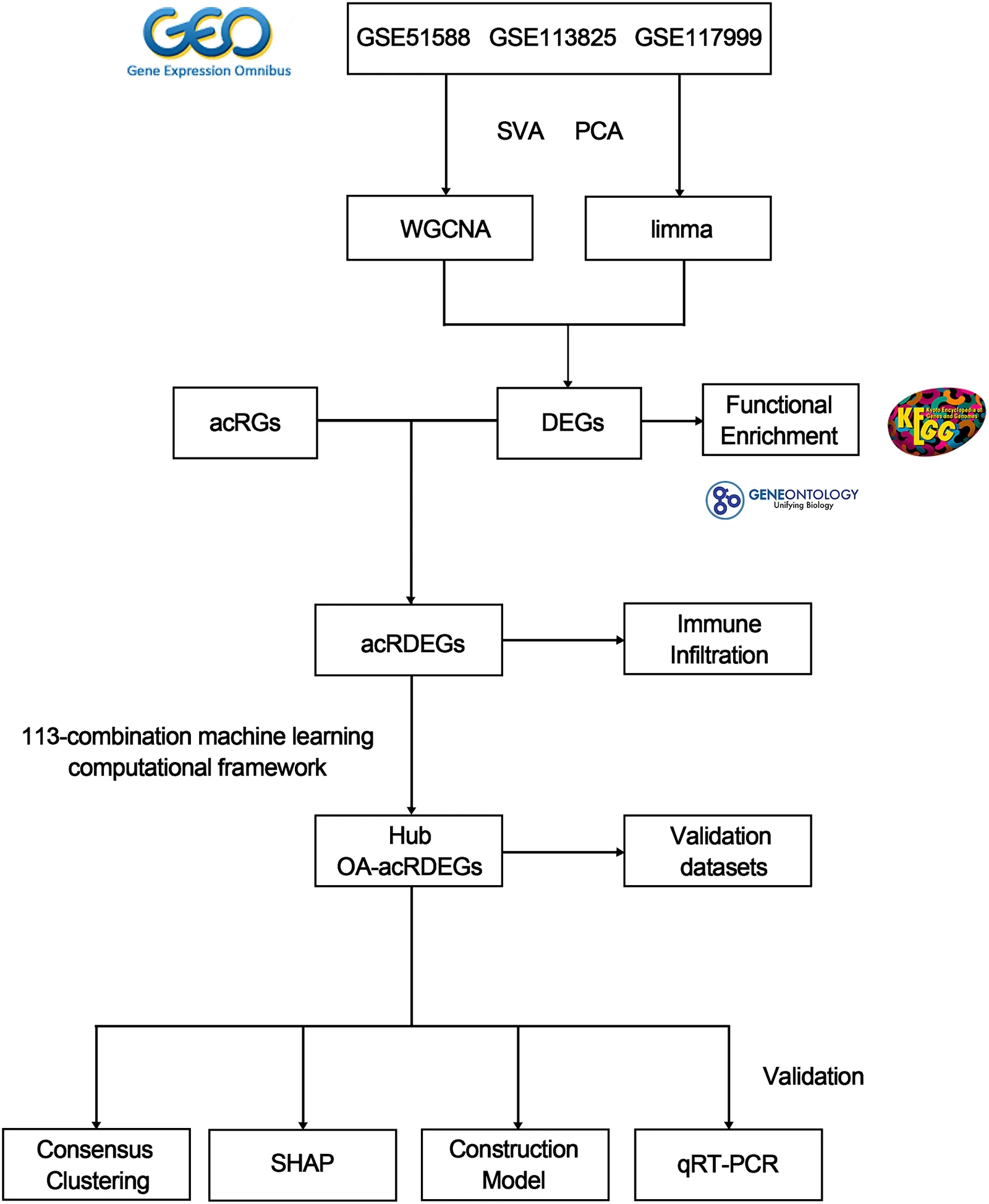
Study workflow. GEO datasets were integrated after surrogate variable analysis (SVA) and principal component analysis (PCA), followed by differential expression analysis, weighted gene co-expression network analysis (WGCNA), functional enrichment, and immune-signature analysis. Osteoarthritis-associated differentially expressed genes overlapping the predefined ac4C-related gene set were screened using 113 machine-learning pipelines and further evaluated by SHAP analysis, model visualization, consensus clustering, and qRT-PCR.

**Fig 2:**
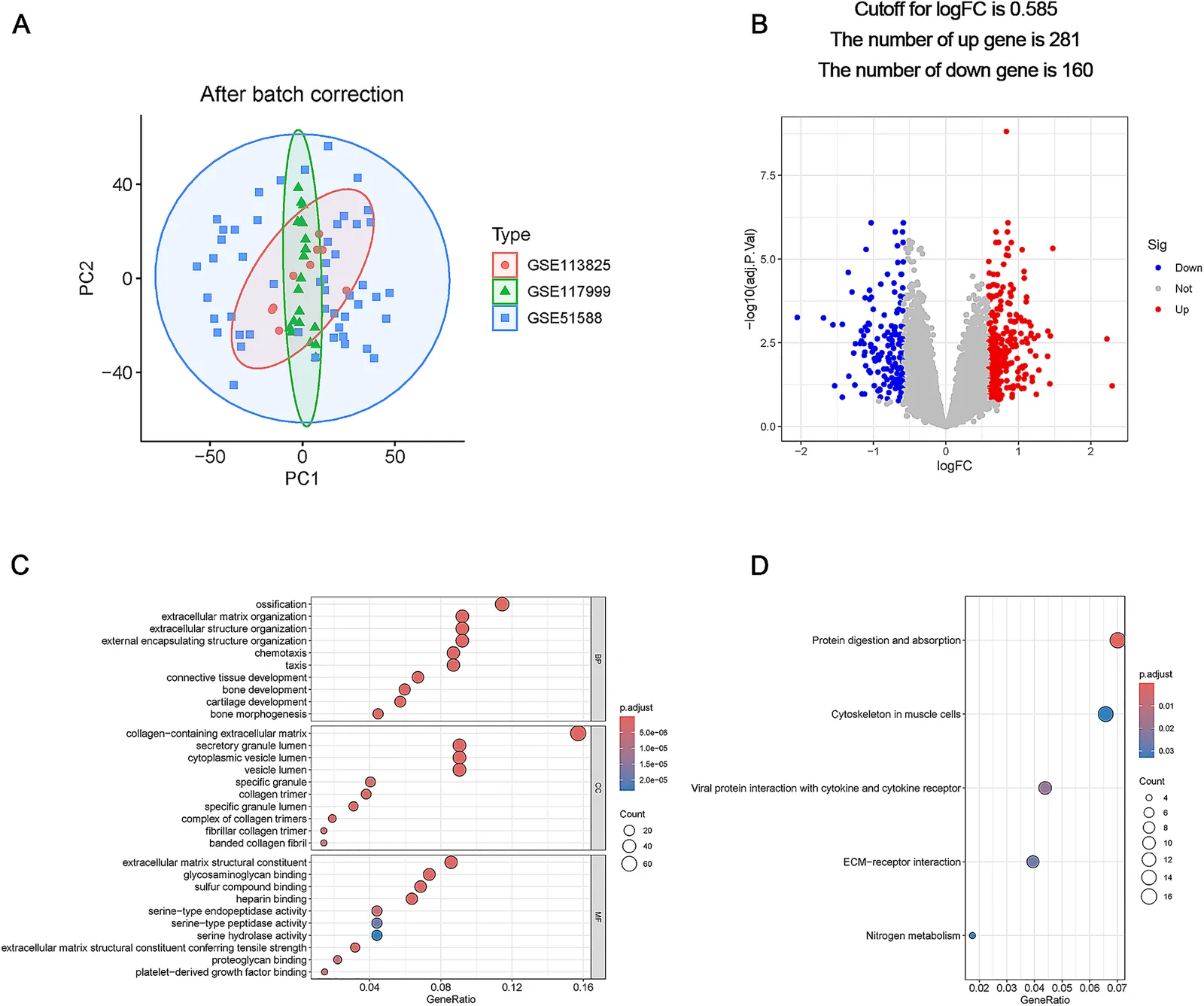
Data preprocessing, differential expression, and functional enrichment analyses. (A) PCA of the integrated expression matrix after ComBat adjustment. Colors and shapes indicate dataset sources. PCA was used as a qualitative diagnostic, and no formal quantitative estimate of batch-associated variance was available. (B) Volcano plot of DEGs using |log2 fold change| > 0.585 and adjusted P < 0.05; red, blue, and grey indicate upregulated, downregulated, and non-significant genes, respectively. (C, D) Gene Ontology (GO) and Kyoto Encyclopedia of Genes and Genomes (KEGG) enrichment analyses. Bubble size represents gene count, and color represents the adjusted P-value. BP, biological process; CC, cellular component; MF, molecular function.

The research design flowchart is shown in Fig 1. PCA was used as a qualitative diagnostic after ComBat adjustment; residual dataset- and tissue-source effects cannot be excluded (Fig 2A). Subsequently, differential expression profiling was performed using thresholds of |logFC| > 0.585 and adjusted P-value < 0.05. A total of 441 DEGs were identified, comprising 281 upregulated and 160 downregulated genes relative to the control group. These results are visualized in the volcano plot (Fig 2B), where significantly dysregulated genes are highlighted.

### Functional enrichment analysis of DEGs

To elucidate the potential mechanisms underlying DEGs in OA, GO and KEGG pathway enrichment analysis were performed using the clusterProfiler software package. The key findings from GO enrichment analysis revealed: Biological processes: ossification, extracellular matrix organization, extracellular structure organization. Cellular component: collagen containing extracellular matrix, secretory granule lumen, cytoplasmic vesicle lumen. Molecular function: extracellular matrix structural constituent, glycosaminoglycan binding, sulfur compound binding. The visualization results of DEGs are shown in Fig 2C. KEGG pathway analysis revealed significant enrichment in protein digestion and absorption, cytoskeleton in muscle cells, viral protein interaction with cytokine and cytokine receptor, ECM−receptor interaction, and nitrogen metabolism, with pathway interconnections depicted in Fig 2D.

### WGCNA

**Fig 3:**
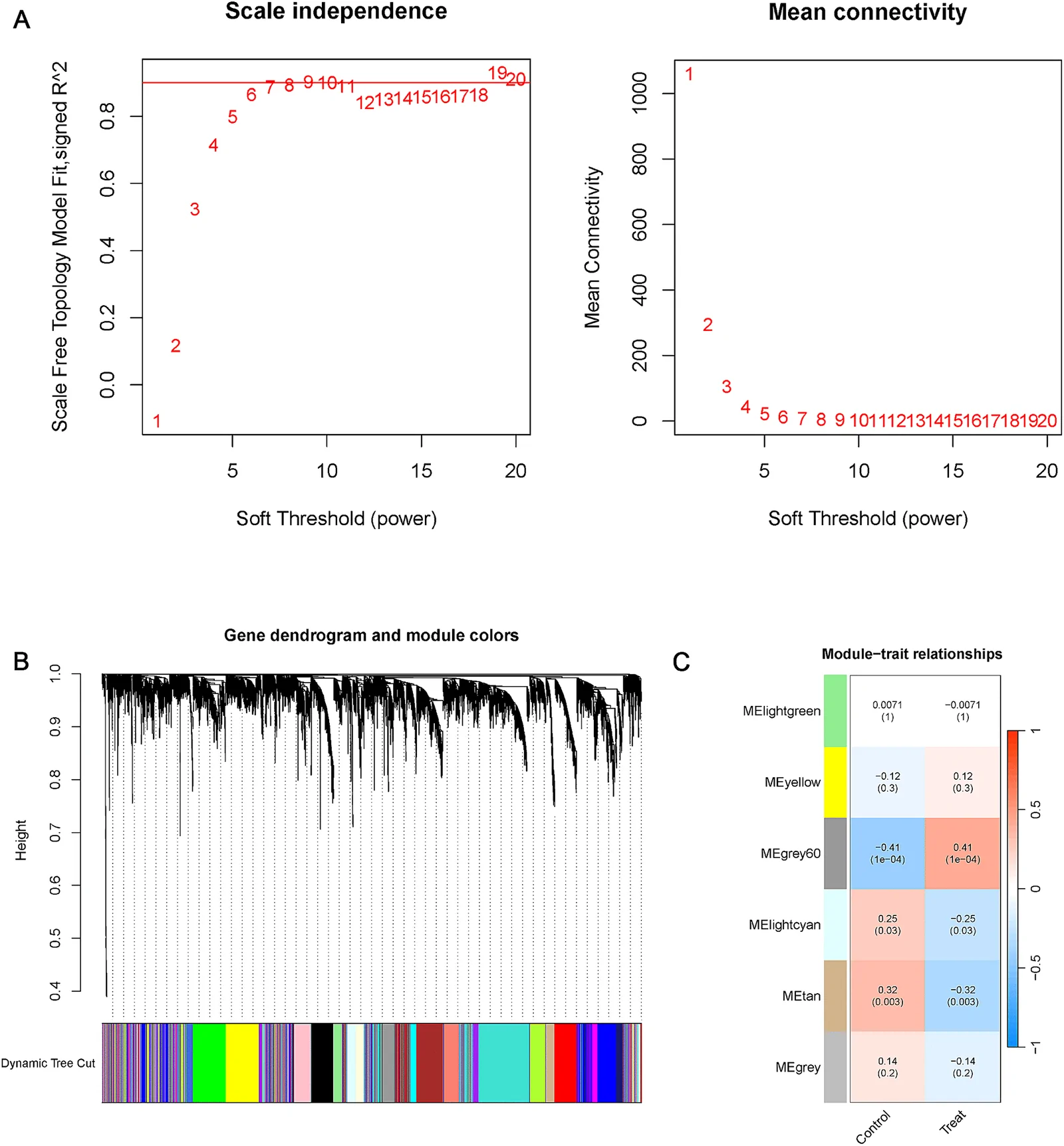
WGCNA. (A) Determine the best soft threshold. The soft threshold value of 6 was determined as the optimal choice for constructing a scale-free network based on the position of the red line (R^2^ = 0.9). (B) Hierarchical clustering dendrogram and dynamic tree-cut module assignment for the 6,061 genes retained after filtering for an across-sample standard deviation greater than 0.5. Each branch represents an individual gene, and the color bar below the dendrogram indicates the corresponding co-expression module. (C) Module–trait relationships between OA and control samples. Red and blue indicate positive and negative correlations, respectively; values represent correlation coefficients, with P-values in parentheses.

Genes with an across-sample standard deviation greater than 0.5 were retained, yielding 6,061 genes for WGCNA. The optimal soft-thresholding power was 6 (R^2^ = 0.9), supporting construction of an approximately scale-free network (Fig 3A). Dynamic tree cutting with a minimum module size of 50 identified six modules (Fig 3B). The grey60 module, comprising 965 genes, showed the strongest association with OA (P = 1 × 10^-4^) and was retained for subsequent analysis (Fig 3C).

### Identification of acRDEGs in osteoarthritis

**Fig 4:**
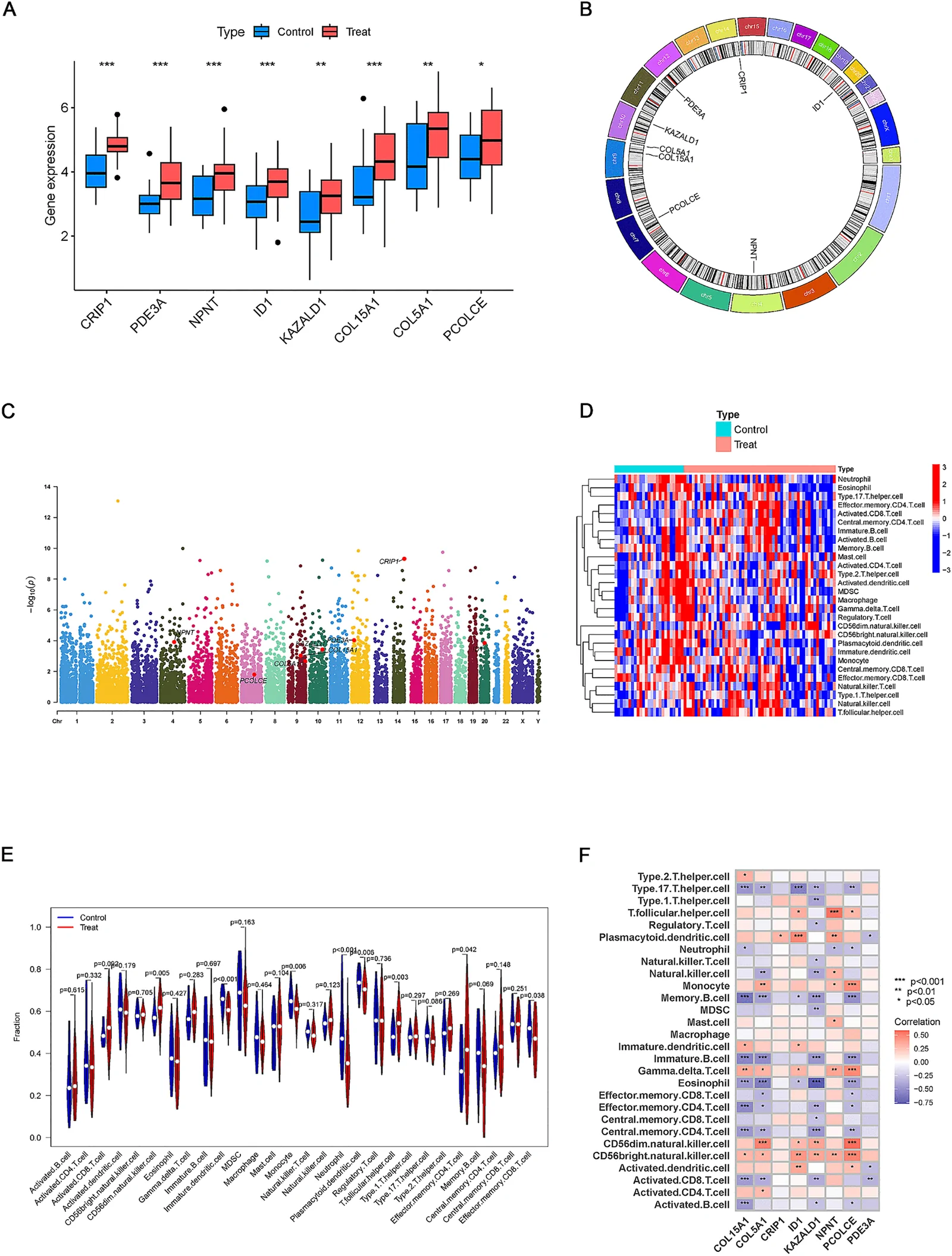
Identification and immune-signature characterization of ac4C-related differentially expressed genes. (A) Expression of 8 acRDEGs in OA samples. (B, C) Association results for the eight acRDEGs, with statistically significant loci highlighted within the chromosomal regions. (D) Heatmap of ssGSEA derived immune cell associated transcriptomic signature scores across samples; red and blue indicate relatively high and low scores, respectively. (E) Violin plots comparing the 28 immune cell associated signature scores between OA and control samples. (F) Correlation analysis between 8 acRDEGs and 28 immune cell types; red and blue indicate positive and negative correlations. (*P < 0.05, **P < 0.01, and ***P < 0.001).

Based on previously published studies, we selected a total of 2135 acRGs for downstream analysis. Using the “VennDiagram” software package, we performed an intersection analysis between DEGs and acRGs, identifying 8 overlapping ac4C-related differentially expressed genes (acRDEGs), all of which were upregulated in OA. (Fig 4A). Chromosomal localization data and their regional distribution are shown in Fig 4B and 4C. This overlap was used only as a candidate filtering step and does not demonstrate direct ac4C modification or NAT10-dependent regulation of these genes in OA.

### Immune associated transcriptomic signature analysis

ssGSEA derived immune cell associated transcriptomic signature scores were higher for CD56dim natural killer cell, T follicular helper cell, and effector-memory CD4 T cell signatures, whereas scores for immature dendritic cell, monocyte, neutrophil, plasmacytoid dendritic cell, and effector memory CD8 T cell signatures were lower in OA samples (Fig 4D and E). Additionally, these 8 acRDEGs correlated with different immune cell types; for instance, PCOLCE showed a positive association with CD56 dim natural killer cells and a negative one with memory B cell, while KAZALD1 was positively linked to CD56 bright natural killer cells and negatively to eosinophil (Fig 4F). These score differences represent enrichment of predefined transcriptional programs and do not directly quantify immune-cell abundance.

### Integration of machine learning algorithms for diagnostic model development

**Fig 5:**
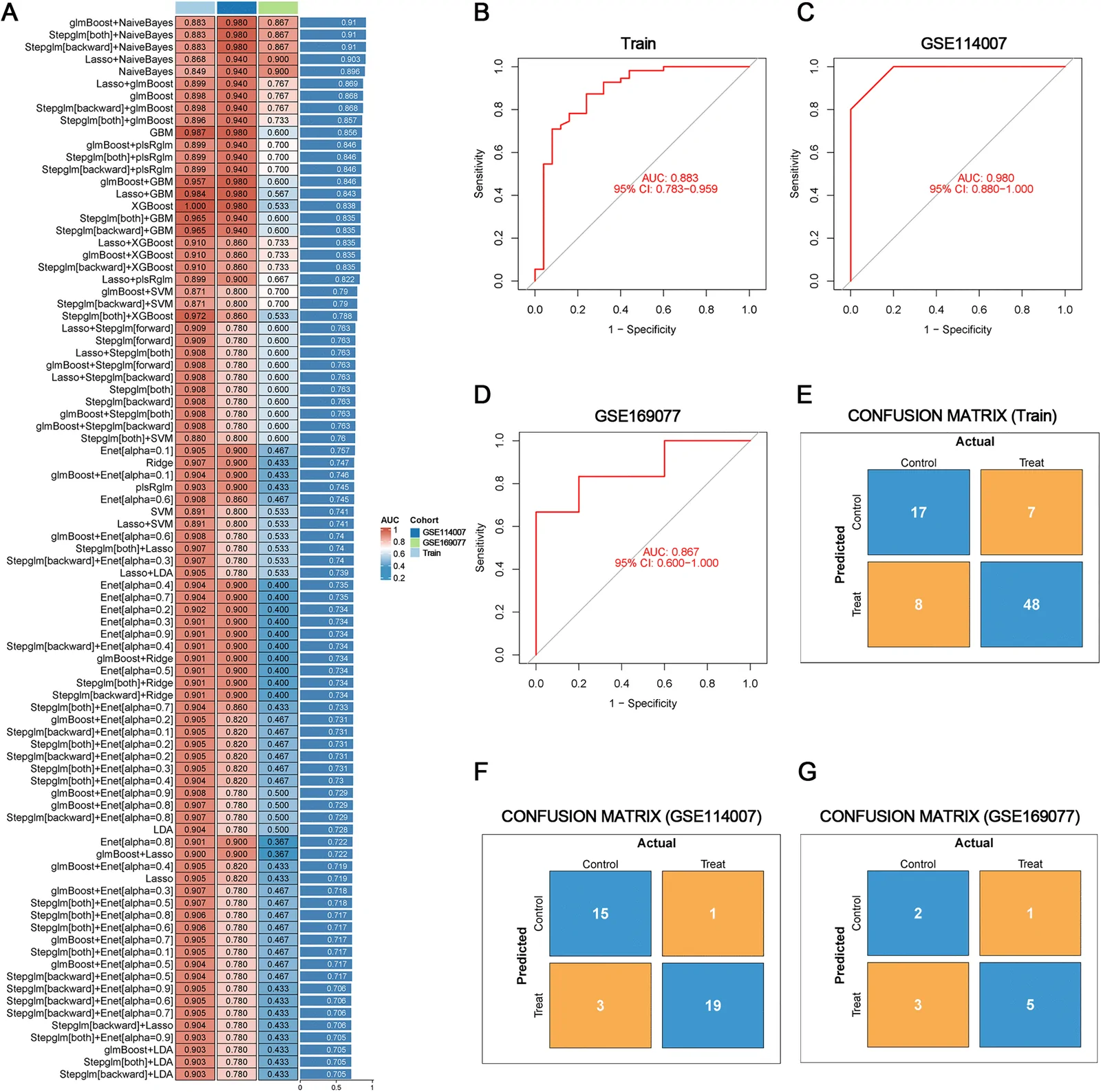
Exploratory multi-cohort machine-learning evaluation. (A) AUC heatmap for 113 candidate configurations in the development cohort, GSE114007, and GSE169077; the final column is the arithmetic mean used for ranking. Three configurations tied at a displayed mean AUC of 0.910, and glmBoost–Naive Bayes was retained as a representative tied configuration. (B–D) ROC curves for the development cohort (n = 80), GSE114007 (n = 38; 20 OA and 18 controls), and GSE169077 (n = 11 pooled arrays). (E–G) Confusion matrices. For GSE114007, TN = 15, FP = 3, FN = 1, and TP = 19. OA/Treat was the positive class. Because both external cohorts contributed to ranking, these panels represent exploratory external evaluation rather than independent validation.

Among the 113 candidate configurations, glmBoost–Naive Bayes, Stepglm[both]–Naive Bayes, and Stepglm[backward]–Naive Bayes shared the highest displayed mean AUC of 0.910 across the development cohort, GSE114007, and GSE169077 (Fig 5A). glmBoost–Naive Bayes was retained as a representative joint-leading configuration for downstream analysis, without claiming superiority over the two tied pipelines. Its AUCs were 0.883 (95% CI, 0.783–0.959), 0.980 (95% CI, 0.880–1.000), and 0.867 (95% CI, 0.600–1.000), respectively (Fig 5B–D). In the development cohort (n = 80), accuracy was 81.25%, sensitivity 87.3%, specificity 68.0%, precision 85.7%, F1 score 86.5%, and balanced accuracy 77.6%. The complete GSE114007 cohort included 20 OA and 18 control samples (n = 38). Its confusion matrix contained 15 true negatives, 3 false positives, 1 false negative, and 19 true positives, corresponding to an accuracy of 89.5%, sensitivity of 95.0%, specificity of 83.3%, precision of 86.4%, F1 score of 90.5%, and balanced accuracy of 89.2%. In GSE169077 (n = 11 pooled samples), the corresponding values were 63.6%, 83.3%, 40.0%, 62.5%, 71.4%, and 61.7% (Fig 5E–G). Because both external cohorts contributed to model ranking, and GSE169077 had a small pooled design, these findings constitute exploratory external evaluation rather than independent validation.

### Validation of Hub OA-acRDEGs

**Fig 6:**
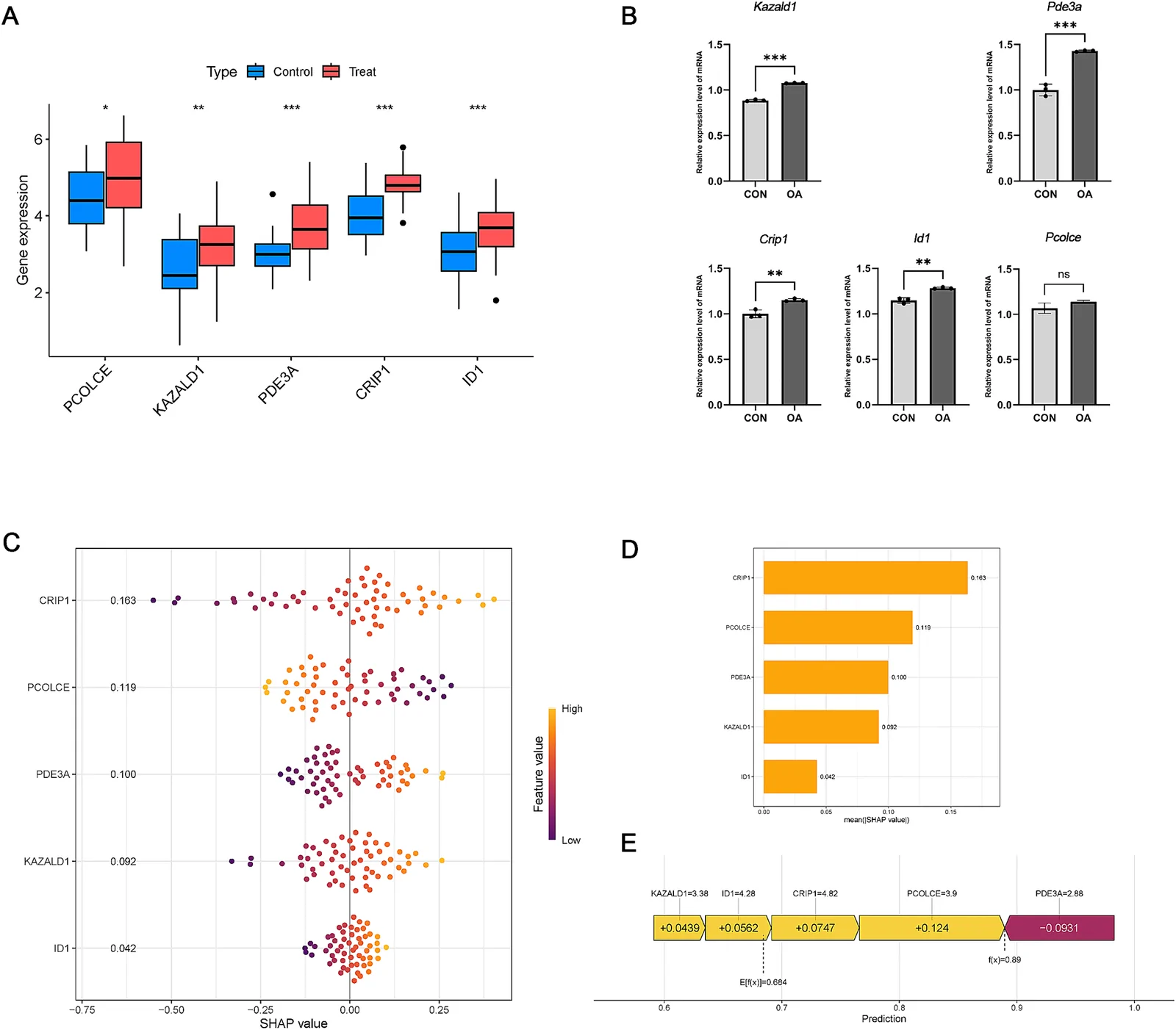
Candidate-gene expression, qRT-PCR assessment, and SHAP interpretation. (A) Expression distributions of the five genes retained in the representative transcriptomic signature. (B) qRT-PCR assessment of all five corresponding mouse orthologs in control and IL-1β-treated primary chondrocytes. Data are presented as mean ± SD from three independent biological replicates per group (n = 3); each biological sample was assayed in three technical wells, which were averaged before analysis. Groups were compared using an unpaired two-sided Student’s t-test. Kazald1, Pde3a, Crip1, and Id1 were significantly increased after IL-1β treatment, whereas Pcolce did not reach statistical significance. ns, not significant; *P < 0.05; **P < 0.01; ***P < 0.001. (C) SHAP summary plot showing signed feature attributions within the fitted model. (D) Mean absolute SHAP values for global model attribution. (E) Force plot for one fitted sample. SHAP values explain the model output and do not demonstrate biological causality or independent biomarker effects.

By integrating the results of multiple algorithms, five hub OA-acRDEGs were identified, including *PCOLCE*, *KAZALD1*, *PDE3A*, *CRIP1*, and *ID1*. All five genes were upregulated in patients with OA compared with the control group (Fig 6A). Further qPCR validation confirmed that the mRNA expression levels of *Kazald1*, *Pde3a*, *Crip1*, and *Id1* were significantly increased in the OA group, whereas *Pcolce* did not show a statistically significant difference between the two groups (Fig 6B). Thus, four of the five computational candidates were supported at the mRNA level in the current IL-1β treated chondrocyte model; *PCOLCE* remains an unconfirmed computational component of the signature.

### SHAP analysis

To further evaluate the contribution of these hub genes to model predictions, SHAP analysis was performed. The SHAP summary plot showed that the five hub genes differed in both the direction and magnitude of their effects on the model output, with a certain degree of inter-sample heterogeneity observed across samples (Fig 6C). Global feature importance ranking based on the mean absolute SHAP values indicated that CRIP1 contributed the most to the model, followed by PCOLCE, PDE3A, KAZALD1, and ID1, with mean values of 0.163, 0.119, 0.100, 0.092, and 0.042, respectively (Fig 6D). At the individual-sample level, SHAP interpretation revealed that KAZALD1, ID1, CRIP1, and PCOLCE made positive contributions to the model output, whereas PDE3A exerted a negative contribution, collectively shifting the predicted value for this sample from the baseline value of 0.684 to 0.89 (Fig 6E).

### Exploratory visualization of the five-gene classification signature

**Fig 7:**
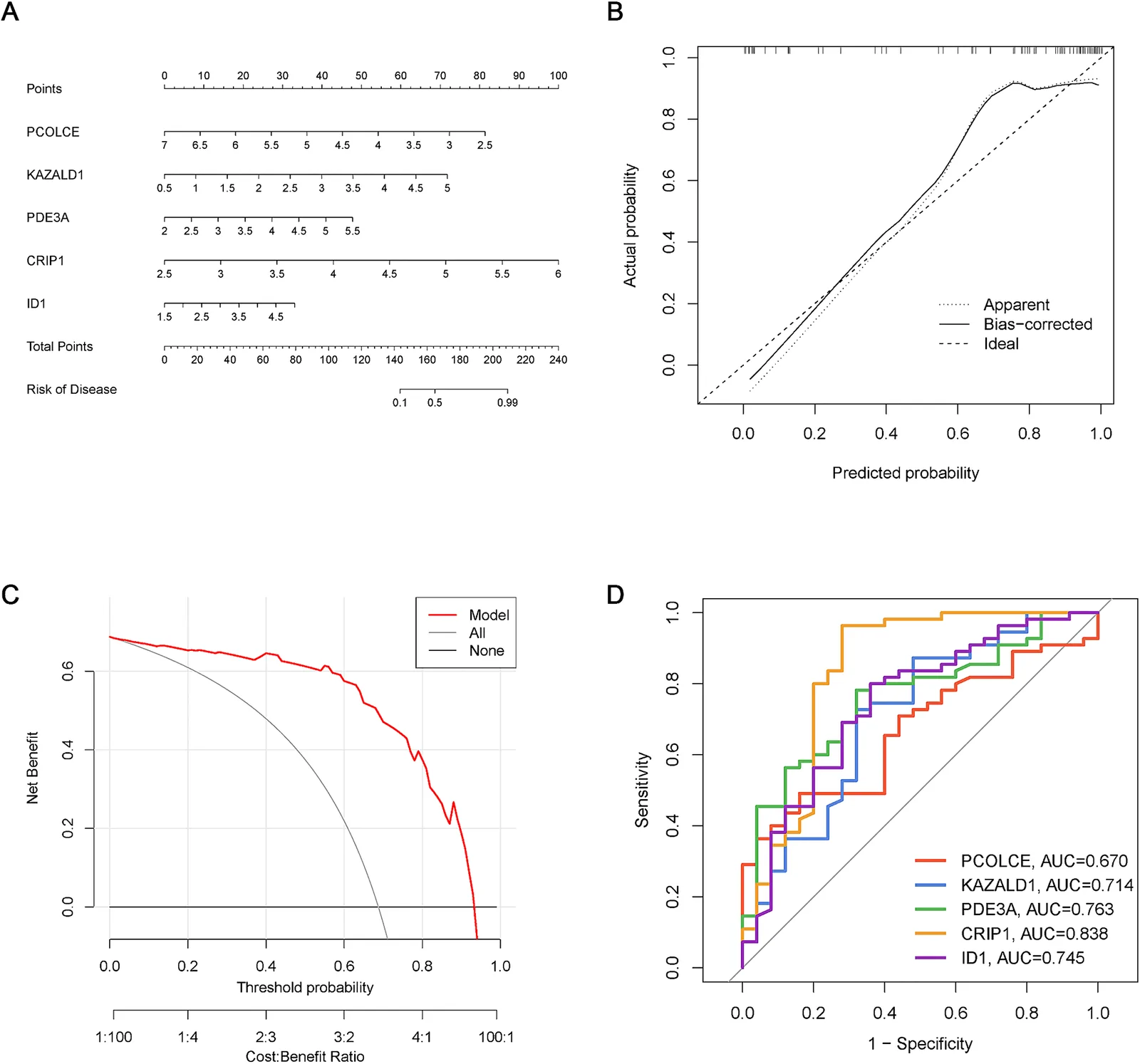
Exploratory visualization and discrimination assessment of the five-gene transcriptomic signature. (A) Nomogram of Hub OA-acRDEGs in the diagnosis of OA patients. (B) Calibration curve used to estimate the predictive accuracy of the nomogram (the closer to the ideal dashed line, the more reliable the result). (C) Net benefit of the clinical decision curve detection model (the further the red line endpoints are from the grey line, the higher the net benefit). (D) ROC curve analysis of Hub OA-acRDEGs.

To provide an exploratory visualization of the classification performance of the five retained genes, we constructed a nomogram based on PCOLCE, KAZALD1, PDE3A, CRIP1, and ID1 (Fig 7A). The nomogram generated an OA classification score within the analyzed cohort. The calibration curve showed that the bias-corrected curve was generally close to the ideal reference line, suggesting apparent agreement between the predicted and observed probabilities in the same dataset (Fig 7B). Decision curve analysis indicated that the model yielded a higher estimated net benefit than the treat-all and treat-none strategies across part of the evaluated threshold-probability range (Fig 7C). However, because both the calibration and decision curve analyses were conducted within the analyzed cohort, these results represent apparent performance and do not establish external calibration, incremental clinical utility, or clinical applicability. Among the five individual genes, CRIP1 showed the highest AUC in the analyzed cohort; nevertheless, this exploratory comparison does not establish CRIP1 as an independently validated diagnostic biomarker (Fig 7D).

### Molecular subtyping of Hub OA-acRDEGs

**Fig 8:**
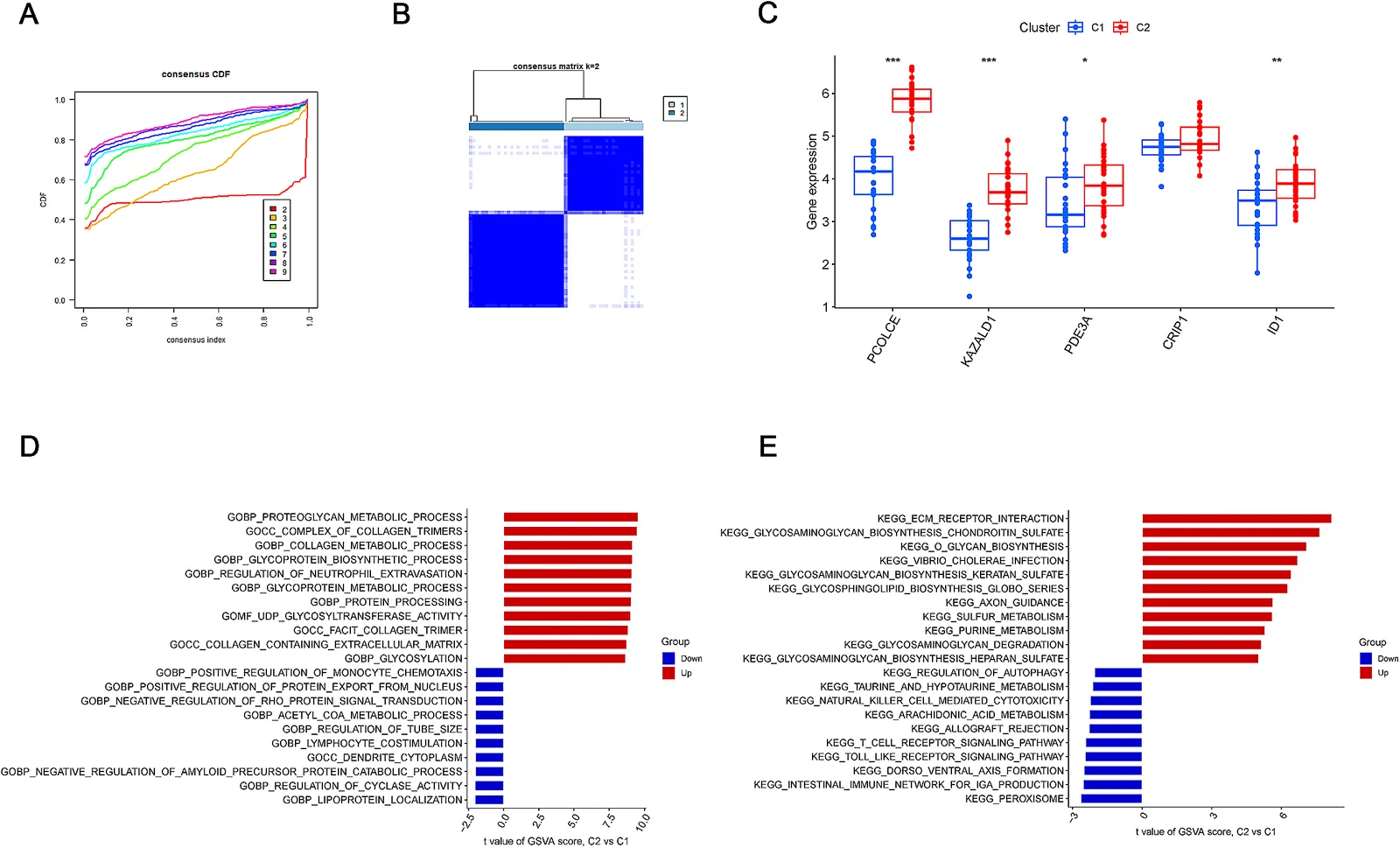
Consensus clustering and pathway-score differences between OA expression clusters. (A) Consensus cumulative distribution function curves for k = 2-9. (B) Consensus matrix for the two-cluster solution; darker blue indicates a higher consensus index. ConsensusClusterPlus was run with 50 resampling iterations, pItem = 0.8, pFeature = 1, k-means clustering, Euclidean distance, and a random seed of 123456. (C) Expression of the five retained genes in C1 (n = 25) and C2 (n = 30); blue and red indicate C1 and C2, respectively. (D, E) Differences in GO- and KEGG-based GSVA scores between C2 and C1. Red indicates higher scores in C2, whereas blue indicates lower scores. *P < 0.05, **P < 0.01; ***P < 0.001.

Based on visual inspection of the CDF curves and consensus matrix, k = 2 showed a relatively clear block structure among the evaluated solutions and was selected for exploratory subtype characterization (Fig 8A). The corresponding consensus matrix revealed strong intra-subtype consistency and significant inter-subtype differences at this clustering level (Fig 8B). This analysis divided the cohort into two subtypes: C1 (n = 25) and C2 (n = 30). Subsequent comparative analysis of hub gene expression revealed subtype-specific characteristics: PCOLCE, KAZALD1, PDE3A, and ID1 were significantly upregulated in the C2 subtype, whereas CRIP1 showed no obvious change in the C2 subtype (Fig 8C). The two cluster solution was not evaluated in an independent dataset and should therefore be regarded as exploratory.

### GSVA enrichment analysis

We investigated GO and KEGG pathway enrichment in OA through the GSVA method. Compared to the C1 group, the C2 group exhibited high enrichment scores in proteoglycan metabolic process, complex of collagen trimers, and collagen metabolic process. At the same time, low enrichment scores were observed in lipoprotein localization, regulation of cyclase activity, and negative regulation of amyloid precursor protein catabolic process (Fig 8D). As indicated by the KEGG pathway enrichment results, compared to the C1 group, the C2 group exhibited high enrichment scores in the following pathways: ECM receptor interaction, glycosaminoglycan biosynthesis chondroitin sulfate, and O glycan biosynthesis. In contrast, peroxisome, intestinal immune network for IGA production, and dorso-ventral axis formation were lower (Fig 8E).

## Discussion

This study identified five OA associated genes within a predefined ac4C related transcript set and retained them in an exploratory machine learning signature. The analysis connects the candidates with ECM related processes, immune associated transcriptomic signatures, and exploratory expression based grouping. qRT-PCR in an IL-1β treated primary mouse chondrocyte model provided partial mRNA level support for four of the five candidates. The study does not directly measure ac4C modification, establish causal gene function, or provide a clinically validated diagnostic test.

One of the main observations in this study is that the functional profile of OA-related genes was strongly enriched in processes associated with ECM remodeling. GO and KEGG analyses consistently highlighted ECM organization, extracellular structure organization, collagen-containing ECM, glycosaminoglycan binding, ossification, and ECM–receptor interaction. These results are in line with the current understanding that OA is characterized by progressive disruption of cartilage matrix homeostasis rather than being solely a consequence of mechanical degeneration [24]. The cartilage ECM is essential for maintaining tissue integrity and biomechanical function, and the loss of balance between matrix synthesis and degradation is a central event in OA development [25]. In this context, the enrichment of collagen- and proteoglycan-related pathways indicates that the genes identified in our study are closely linked to structural changes in diseased joint tissues. The enrichment of ossification related terms is also notable, because OA progression is often accompanied by osteochondral remodeling, calcification, and subchondral bone changes [26]. However, the interpretation of the ac4C relationship needs to be cautious. The 2135 gene lists used in our analysis were derived from previously published studies that classified them as ac4C related gene sets based on ac4C RIP seq comparisons between wild-type and NAT10 deficient HeLa cells. No direct ac4C profiling or NAT10 perturbation experiments were performed in OA tissues or chondrocytes. Therefore, the overlap between this list and differentially expressed genes in OA only identified genes related to the ac4C related transcript set reported in this study, but did not demonstrate the presence of ac4C modification or ac4C dependent regulation of these genes in OA.

An important aspect of this work is the focus on ac4C-related regulation. N4-acetylcytidine (ac4C) is a conserved RNA modification that has been reported to affect mRNA stability and translation efficiency [27]. However, compared with other epitranscriptomic modifications, its role in OA has remained largely unclear. Our results suggest that ac4C-related genes may be involved in the molecular network underlying OA. This is plausible given that chondrocytes in the OA micro environment are exposed to persistent inflammatory stimulation, oxidative stress, abnormal mechanical loading, and senescence related changes [28]. These stressors require dynamic and finely controlled post transcriptional regulation, and RNA modifications may contribute to this adaptive process. Although our study did not directly measure ac4C abundance or identify acetylated transcripts, the identification of OA-acRDEGs supports the hypothesis that ac4C-related pathways are associated with OA biology. This provides a basis for future mechanistic studies on how ac4C modification influences cartilage homeostasis and degeneration.

Another relevant finding is the association between the identified candidate genes and immune-related transcriptomic features. OA is increasingly recognized as a whole-joint disease with a low-grade inflammatory component [29]. In the present study, immune-associated signature scores differed between OA and control samples; however, these differences may reflect altered immune-related transcriptional activity, tissue composition, or stromal expression and do not establish changes in immune-cell abundance. The associations between the candidate genes and immune-related signature scores indicate transcriptomic co-variation but do not demonstrate direct immunoregulatory functions. This distinction is particularly important because the development cohort combines cartilage and subchondral-bone samples and the 28 signatures were not developed specifically for OA tissue. Different deconvolution methods, including CIBERSORT, xCell, and MCP-counter, use distinct signatures and assumptions and may produce different estimates. Accordingly, the present findings are hypothesis-generating and require confirmation using independent deconvolution frameworks, single-cell or spatial profiling, histology, or flow cytometry [30,31].Consistent with this broader immune context, a recent study integrating bulk and single-cell transcriptomic data identified shared M0 macrophage-associated gene signatures across osteoarthritis and osteomyelitis, further highlighting the intersection between joint degeneration, macrophage-associated inflammation, and bone inflammatory disease [32].

The five hub OA-acRDEGs identified in this study have biological features that support their potential relevance to OA. PCOLCE is involved in procollagen processing and collagen maturation, indicating a close relationship with ECM organization and tissue remodeling [33]. Its dysregulation may reflect altered collagen turnover in OA cartilage or surrounding joint tissues. KAZALD1 has been associated with skeletal development and extracellular matrix regulation. The upregulation of Kazald1 in OA patients may reflect a compensatory protective response to cartilage damage and aberrant TGF-β signaling, rather than a detrimental effect per se. Although Kazald1 has been reported to inhibit chondrocyte fibrosis and favor hyaline cartilage regeneration, its endogenous increase in OA may be insufficient to counterbalance the persistent catabolic and fibrotic milieu of the diseased joint [34]. PDE3A is a phosphodiesterase that regulates cyclic nucleotide signaling and may affect inflammatory responses, cellular metabolism, and stress adaptation [35]. Because cAMP and cGMP related pathways are involved in cell survival and inflammatory regulation, PDE3A may influence chondrocyte behavior under pathological conditions [36]. Previous studies have demonstrated that CRIP1 exacerbates osteoarthritis progression by functioning as a scaffold protein that mediates the interaction between UBE3A and MFGE8, leading to ubiquitin proteasome dependent degradation of MFGE8 and subsequent activation of the NF-κB pathway in chondrocytes [37]. Moreover, CRIP1 showed the largest mean absolute SHAP value within the fitted multigene model and the highest single-gene AUC in the analyzed cohort. It is therefore considered an informative component of the candidate five-gene signature rather than a validated standalone biomarker. ID1 is a transcriptional regulator involved in cell proliferation and differentiation, and its dysregulation may contribute to the abnormal phenotypic transition of chondrocytes during OA progression. Previous studies have shown that ID1 is upregulated through the TGF-β/SMAD1/5/9 pathway, which represents the detrimental arm of TGF-β signaling in OA chondrocytes, suggesting that ID1 may serve as a marker of pathogenic TGF-β activity in OA [38]. Overall, these genes converge on pathways related to matrix remodeling, cellular regulation, and pathological differentiation, which are all central to OA pathogenesis.

The machine learning results provide an exploratory, interpretable five gene signature rather than evidence of clinical readiness. The three leading configurations were tied at the displayed precision, and glmBoost–Naive Bayes was retained as a representative. Moreover, GSE114007 and GSE169077 contributed to the mean AUC ranking; consequently, their performance estimates are susceptible to selection related optimism and cannot be regarded as fully independent external validation. Although evaluation of the complete GSE114007 cohort (n = 38) yielded favorable discrimination and threshold-based performance, the generalizability of the model remains limited by the modest cohort size, cross-platform heterogeneity, mixed tissue sources in the development cohort, and the lower accuracy and specificity observed in the small pooled GSE169077 cohort, which also had a wide AUC confidence interval. The archived code allowed recovery of the global random seed and major algorithm-specific settings; however, the original software-version environment, saved fold assignments, fold-specific seed vectors, complete model-configuration file, and several realized data-dependent tuning values, including lambda, mstop, the final number of GBM trees, and XGBoost nrounds, were not preserved. Moreover, scaling and feature selection were performed on the full development matrix rather than repeated within each resampling fold, which may have introduced information leakage and model-selection optimism and may limit exact numerical reproduction of all 113 pipelines. A definitive assessment requires a prospectively specified pipeline, training-only model selection, and one time evaluation in a larger untouched multicenter cohort.

Consensus clustering suggested two candidate expression groups with distinct pathway activity scores. Because the clusters were derived from only five genes in 55 OA samples and were not reproduced in an independent dataset, they should be regarded as hypothesis generating groups rather than validated molecular subtypes or a basis for individualized therapy.

The qRT-PCR experiment comprised three independent biological replicates per group, with each biological sample measured in technical triplicate. *Kazald1*, *Pde3a*, *Crip1*, and *Id1* showed significant increases after IL-1β treatment, whereas *Pcolce* did not reach statistical significance. These findings provide partial mRNA-level support for four of the five computational candidates, but they should not be interpreted as validation of an ac4C dependent mechanism. The small biological sample size, use of an acute mouse chondrocyte inflammatory model, absence of protein level confirmation, and lack of human cartilage or independent in vivo validation limit the experimental evidence. *PCOLCE* expression may vary according to biological context, tissue type, or disease stage and therefore requires further investigation.

Several limitations should be considered when interpreting these findings. First, the computational analyses were retrospective and based on public datasets generated across different platforms and tissue sources. The development cohort combined cartilage and subchondral-bone samples, while detailed clinical variables such as age, sex, body mass index, medication use, and disease stage were incomplete. Residual batch effects, tissue-composition differences, donor dependence, and unmeasured clinical heterogeneity may therefore have influenced the results. No formal quantitative before-versus-after batch-variance metric was retained, and the fixed WGCNA variability filter of standard deviation > 0.5 was not evaluated in a sensitivity analysis. In addition, GSE114007 and GSE169077 were used during the ranking of 113 candidate pipelines, which may introduce selection-related optimism; their performance should therefore be regarded as exploratory external evaluation rather than independent validation. Second, the predefined ac4C-related gene set originated from an ac4C-RIP-seq comparison in wild-type and NAT10-deficient HeLa cells. Its overlap with OA-associated transcripts identifies candidate ac4C-associated genes but does not demonstrate direct ac4C modification or NAT10-dependent regulation in OA. Likewise, ssGSEA-derived immune-associated signature scores do not directly quantify immune-cell abundance, and no orthogonal deconvolution method was applied. The two-cluster solution was not independently reproduced. Experimental assessment was limited to qRT-PCR with three biological replicates per group; four genes showed nominally significant increases, whereas Pcolce was not significant. The absence of multiplicity correction, protein-level confirmation, human-cartilage validation, in vivo experiments, and functional perturbation means that these results provide only preliminary mRNA-level support. Future studies should use a prospectively specified, leakage-controlled pipeline with a locked independent test cohort, archive complete software environments and resampling records, directly profile ac4C and perturb NAT10, perform orthogonal immune validation, and confirm the candidate genes and clusters through protein-level, functional, human-tissue, and in vivo studies.

## Conclusions

In conclusion, *PCOLCE*, *KAZALD1*, *PDE3A*, *CRIP1*, and *ID1* were retained in an exploratory OA associated transcriptomic signature. qRT-PCR provided partial mRNA level support for *KAZALD1*, *PDE3A*, *CRIP1*, and *ID1*, whereas *PCOLCE* was not significant in the current IL-1β treated chondrocyte model. Three candidate pipelines shared the highest displayed mean AUC, and glmBoost-Naive Bayes was retained as a representative configuration. Because both external cohorts contributed to model ranking, external performance is exploratory rather than independent validation. The immune signature associations, nomogram, and two cluster solution are hypothesis generating. Direct ac4C measurement, complete computational documentation, larger independent biological validation, protein and functional experiments, and prospective evaluation are required before mechanistic or clinical conclusions can be drawn.

## Supporting information

S1 Table Characteristics and analytical roles of the GEO datasets. Summary of the sample composition, tissue sources, experimental platforms, study characteristics, and analytical roles of GSE51588, GSE113825, GSE117999, GSE114007, and GSE169077.
(PDF)

S2 Table Machine-learning framework, parameter settings, and reproducibility information. Summary of the study design, cohort allocation, preprocessing procedures, machine-learning algorithms, tuning strategies, model-selection criteria, performance metrics, software implementation, code availability, SHAP analysis, and consensus clustering settings.
(PDF)

S3 Table Primer sequences used for qRT-PCR validation. Forward and reverse primer sequences for mouse Actb, Pcolce, Kazald1, Pde3a, Crip1, and Id1.
(PDF)
