# Supplementary material for: Identification of transcriptomic signatures associated with an ac4C related gene set and candidate expression based clusters in osteoarthritis through integrative bioinformatics
Source: PLoS One. 2026 Sep 24;21(9):e0359336. doi: 10.1371/journal.pone.0359336 (PMC13606951; doi:10.1371/journal.pone.0359336)
Supplement: Supplementary file 1 [file pone.0359336.s001.pdf]

Supplementary Table S1: Characteristics and analytical roles of the GEO datasets

GEO series	Deposited composition	Samples used	Tissue/source	Platform	Design and key characteristics	Analytical role
GSE51588	40 OA and 10 non-OA samples from 25 donors	50 arrays	Human knee subchondral bone	GPL13497; Agilent whole-human-genome microarray	Lateral and medial tibial-plateau samples were obtained from the same donors; within-donor dependence is possible.	Development cohort
GSE113825	5 OA and 5 non-OA cartilage specimens at gene level; corresponding exon-level profiles also deposited	10 gene-level samples	Human knee articular cartilage	GPL22516/GPL22517; Affymetrix Human OElncRNAs array	Severe OA cartilage versus non-OA cartilage; unpaired comparison.	Development cohort
GSE117999	12 OA and 12 non-OA specimens deposited; four samples excluded in the source study	10 OA and 10 non-OA retained	Human knee articular cartilage	GPL20844; Agilent SurePrint G3 Human GE v3 microarray	End-stage OA from total knee arthroplasty versus non-OA cartilage from arthroscopic partial meniscectomy.	Development cohort
GSE114007	20 OA and 18 non-OA cartilage specimens	All 38 samples	Human knee articular cartilage	GPL11154/GPL18573; Illumina HiSeq 2000 and NextSeq 500 RNA-seq	Two sequencing-platform batches; all deposited OA and control samples were included.	External evaluation cohort; included in mean-AUC ranking
GSE169077	6 OA pools and 5 non-OA pools	11 pooled arrays	Human knee articular cartilage	GPL96; Affymetrix Human Genome U133A Array	Each array represents RNA pooled from five individuals.	External evaluation cohort; included in mean-AUC ranking

Note: OA, osteoarthritis; GEO, Gene Expression Omnibus. Dataset characteristics were checked against the corresponding GEO accession records. GSE51588 contains repeated anatomical samples from donors, and GSE169077 arrays represent pooled RNA.
