# Supplementary material for: Identification of transcriptomic signatures associated with an ac4C related gene set and candidate expression based clusters in osteoarthritis through integrative bioinformatics
Source: PLoS One. 2026 Sep 24;21(9):e0359336. doi: 10.1371/journal.pone.0359336 (PMC13606951; doi:10.1371/journal.pone.0359336)
Supplement: Supplementary file 2 [file pone.0359336.s002.pdf]

Supplementary Table S2: Machine-learning framework, tuning strategy, software implementation, and reproducibility record

Section	Item	Verified implementation	Interpretation / limitation
**Study design**	Prediction target	Cross-sectional classification of OA versus non-OA joint-tissue samples.	Not prognosis or incident-risk prediction.
**Study design**	Candidate feature set	Eight OA DEGs overlapping the predefined 2,135-gene ac4C-related transcript set.	Candidate filtering only; direct ac4C modification in OA was not measured.
**Cohorts**	Development cohort	GSE51588, GSE113825, and GSE117999; nominal n=80.	Mixed cartilage and subchondral-bone sources.
**Cohorts**	External evaluation cohorts	GSE114007 (n=38) and GSE169077 (n=11 pooled arrays).	Both contributed to mean-AUC ranking and were not untouched holdout tests.
**Pipeline**	Algorithms	Lasso, Ridge, Stepglm, XGBoost, RF, Elastic Net, plsRglm, GBM, Naive Bayes, LDA, glmBoost, and SVM.	Arranged into 113 two-stage feature-selection/classification configurations.
**Preprocessing**	Scaling	The development matrix was centered and scaled as a whole; each external cohort was centered and scaled separately.	Scaling was not performed within resampling folds.
**Resampling**	Overall strategy	Algorithm-specific internal cross-validation or fixed settings were used; no single uniform resampling scheme was applied to all algorithms.	A fully nested, fold-contained pipeline was not implemented.
**Tuning**	Lasso / Ridge / Elastic Net	Binomial glmnet models used 10-fold cross-validation and lambda.min. Alpha was fixed at 1 for Lasso and 0 for Ridge; Elastic Net alpha was read from the model-configuration list.	The configuration file containing the tested Elastic Net alpha values was not included in the supplied archive.
**Tuning**	Stepglm	AIC-based stepwise logistic regression using the configuration-specified direction (forward, backward, or both).	No separate hyperparameter grid was used.
**Tuning**	glmBoost	The stopping iteration was selected by cross-validated risk using mboost::cvrisk; final mstop=max(selected mstop, 40).	The data-dependent final mstop was not exported in the supplied archive.
**Tuning**	Random forest	randomForestSRC::rfsrc with ntree=1,000 and nodesize=5; importance, proximity, and forest objects were retained during execution.	These were fixed settings rather than grid-searched values.
**Tuning**	GBM	Initial model: 10,000 trees, interaction.depth=3, n.minobsinnode=10, shrinkage=0.001, and 10-fold CV. Final n.trees was selected by minimum CV error.	The selected tree number was data-dependent and was not exported separately.
**Tuning**	XGBoost	Five-fold CV selected nrounds from up to 10 rounds using minimum test log-loss; max.depth=2, eta=1, and nthread=2.	The final data-dependent nrounds value was not exported separately.
**Tuning**	SVM / Naive Bayes	e1071::svm and e1071::naiveBayes were fitted with package-default settings; probability output was enabled for SVM.	No explicit tuning grid was coded.
**Tuning**	LDA	caret::train(method="lda") with cross-validation specified through trainControl(method="cv").	The fold number was not explicitly set in the script.
**Tuning**	plsRglm	A cv.plsRglm call with nt=10 was generated, followed by a sparse logistic plsRglm fit.	The optimized component count was not explicitly passed to the final fitted model.
**Model selection**	Ranking rule	Arithmetic mean AUC across the development cohort, GSE114007, and GSE169077.	May introduce selection-related optimism.
**Model selection**	Joint-leading configurations	glmBoost-Naive Bayes, Stepglm[both]-Naive Bayes, and Stepglm[backward]-Naive Bayes; displayed mean AUC=0.910.	No superiority was claimed among the tied configurations.
**Final model**	Retained representative	glmBoost-Naive Bayes with PCOLCE, KAZALD1, PDE3A, CRIP1, and ID1.	Used for downstream SHAP and nomogram displays.
**Classification**	Threshold / class assignment	A probability threshold of >0.5 was explicitly coded for glm, plsRglm, GBM, and XGBoost; other algorithms used model-native class predictions.	The retained Naive Bayes model used its native class prediction.
**Performance**	Development cohort	AUC 0.883 (95% CI, 0.783-0.959); accuracy 81.25%; sensitivity 87.3%; specificity 68.0%; precision 85.7%; F1 86.5%; balanced accuracy 77.6%.	TN=17, FP=8, FN=7, TP=48.
**Performance**	GSE114007	AUC 0.980 (95% CI, 0.880-1.000); accuracy 89.5%; sensitivity 95.0%; specificity 83.3%; precision 86.4%; F1 90.5%; balanced accuracy 89.2%.	TN=15, FP=3, FN=1, TP=19.
**Performance**	GSE169077	AUC 0.867 (95% CI, 0.600-1.000); accuracy 63.6%; sensitivity 83.3%; specificity 40.0%; precision 62.5%; F1 71.4%; balanced accuracy 61.7%.	TN=2, FP=3, FN=1, TP=5; pooled arrays.

**Reproducibility**	Global random seed	set.seed(123) was called before the feature-selection loop and again before the model-fitting loop.	Fold-specific seed vectors and saved fold assignments were not archived.
**Reproducibility**	Key R packages	randomForestSRC, glmnet, plsRglm, gbm, caret, mboost, e1071, MASS, xgboost, pROC, ComplexHeatmap, RColorBrewer, openxlsx, seqinr, plyr, BART, and snowfall.	Package names were verified from MLmodel.R.
**Reproducibility**	R and package versions	All analyses were performed using R version 4.3.3. No sessionInfo(), packageVersion() output, renv.lock, or equivalent package-environment file was preserved.	The R version is documented, but the exact package-version environment cannot be reconstructed reliably.
**Reproducibility**	Optimized values	Lambda, mstop, final GBM tree count, and XGBoost nrounds were calculated during execution.	Their realized values were not exported in the supplied code archive.
**Reproducibility**	Fold-specific feature selection	Candidate features were selected on the full scaled development matrix before model fitting.	Feature selection was not repeated within each validation fold; leakage-related optimism cannot be excluded.
**Code availability**	Public repository	Analysis scripts are deposited in Zenodo (DOI: 10.5281/zenodo.21642964).	The repository improves transparency, but exact reproduction also requires the original input matrices, configuration list, and software environment.
**SHAP**	Implementation details	Mean absolute SHAP values and signed sample-level attributions were retained.	The exact estimator wrapper, background dataset, package version, and stability analyses were not archived.
**Clustering**	Consensus settings	ConsensusClusterPlus with k=2-9, reps=50, pItem=0.8, pFeature=1, k-means clustering, Euclidean distance, and seed=123456.	The two-cluster solution remains exploratory and was not independently reproduced.

Footnote: Implementation details were verified directly from the archived R scripts. Data-dependent values that were calculated during execution but not exported in the supplied archive were not reconstructed. AUC, area under the receiver operating characteristic curve; CI, confidence interval; DEG, differentially expressed gene; GBM, gradient boosting machine; LDA, linear discriminant analysis; OA, osteoarthritis; RF, random forest; SHAP, Shapley Additive exPlanations; SVM, support vector machine.
