# Supplementary material for: Identification of transcriptomic signatures associated with an ac4C related gene set and candidate expression based clusters in osteoarthritis through integrative bioinformatics
Source: PLoS One. 2026 Sep 24;21(9):e0359336. doi: 10.1371/journal.pone.0359336 (PMC13606951; doi:10.1371/journal.pone.0359336)
Supplement: Supplementary file 3 [file pone.0359336.s003.pdf]

Supplementary Table S3: Primer sequences

1 **Mus musculus-Actb actin**
Forward:5′-CCACTGTCGAGTCGCGTCC-3′
Reverse:5′-ATTCCCACCATCACACCCTGG-3′

2 **Mus musculus-Pcolce**
Forward:5′- CCCCAACTACACGAGACCTG-3′
Reverse:5′- GACCTCCAGAGCATCGTAGC-3′

3 **Mus musculus-Kazald1**
Forward:5′-ATGTGACCGGACAGGATGTG-3′
Reverse:5′-AGGTCCACCCCTAAACTGC-3′

4 **Mus musculus-Pde3a**
Forward:5′-AGAGATTCCGGGGTGGAAGA-3′
Reverse:5′-TGATGCTGGTTCCTGACTGG-3′

5 **Mus musculus-Crip1**
Forward:5′-GAGCATGAAGGCAAGCCCTA-3′
Reverse:5′-AGAGTTTCCACAACCTCGGTC-3′

6 **Mus musculus-Id1**
Forward:5′-GGTACTTGGTCTGTCGGAGC-3′
Reverse:5′-GCAGGTCCCTGATGTAGTCG-3′
